# Proximity tracking using ultra-wideband technology for equine social behaviour research

**DOI:** 10.1038/s41598-024-60805-0

**Published:** 2024-04-30

**Authors:** Laura Torres Borda, Peter Roth, Jennifer Lumetzberger, Ulrike Auer, Florien Jenner

**Affiliations:** 1 Equine Surgery Unit, Department of Companion Animals and Horses, University of Veterinary Medicine Vienna, Veterinaerplatz 1, 1210 Vienna, Austria; 2 Computational Medicine, Department of Biomedical Sciences, University of Veterinary Medicine Vienna, Vienna, Austria; 3 Anaesthesiology and Perioperative Intensive Care Medicine Unit, Department of Companion Animals and Horses, University of Veterinary Medicine Vienna, Vienna, Austria

**Keywords:** Social behaviour, Social proximity, Social interactions, Ultra-wideband, Affiliative, Agonistic, Sociopositive, Socionegative, Behavioural methods, Animal behaviour

## Abstract

Sociopositive interactions with conspecifics are essential for equine welfare and quality of life. This study aimed to validate the use of wearable ultra-wideband (UWB) technology to quantify the spatial relationships and dynamics of social behaviour in horses by continuous (1/s) measurement of interindividual distances. After testing the UWB devices’ spatiotemporal accuracy in a static environment, the UWB measurement validity, feasibility and utility under dynamic field conditions was assessed in a group of 8 horses. Comparison of the proximity measurements with video surveillance data established the measurement accuracy and validity (r = 0.83, p < 0.0001) of the UWB technology. The utility for social behaviour research was demonstrated by the excellent accordance of affiliative relationships (preferred partners) identified using UWB with video observations. The horses remained a median of 5.82 m (95% CI 5.13–6.41 m) apart from each other and spent 20% (median, 95% CI 14–26%) of their time in a distance ≤ 3 m to their preferred partner. The proximity measurements and corresponding speed calculation allowed the identification of affiliative versus agonistic approaches based on differences in the approach speed and the distance and duration of the resulting proximity. Affiliative approaches were statistically significantly slower (median: 1.57 km/h, 95% CI 1.26–1.92 km/h, p = 0.0394) and resulted in greater proximity (median: 36.75 cm, 95% CI 19.5–62 cm, p = 0.0003) to the approached horse than agonistic approaches (median: 3.04 km/h, 95% CI 2.16–3.74 km/h, median proximity: 243 cm, 95% CI 130–319 cm), which caused an immediate retreat of the approached horse at a significantly greater speed (median: 3.77 km/h, 95% CI 3.52–5.85 km/h, p < 0.0001) than the approach.

## Introduction

Social behaviour is an essential contributor to and a key indicator of equine welfare and quality of life^1–7^.

As gregarious animals that live in stable social groups with strong, lasting bonds between individuals and stable dyadic interaction patterns, horses have highly developed social cognitive abilities and a complex behavioural repertoire for communication, including a range of nuanced visual signals that allow other animals to gauge the intentions of the sender^7–19^. Horses are capable of cross-modal individual recognition using visual, auditory, and olfactory cues even after a year's absence ^8,9,20–26^. In addition, through long-term memorization of past interactions and different degrees of social bonds horses can recognize their fellow group members and discern their individual social status relative to their own^8–10,12,20,21,24–27^. Furthermore, they can predict the outcome of encounters with familiar individuals, adjust to each other's behavioural responses and increase affiliative behaviour after a conflict, thus reducing social tension^5,12,16,17,19,28^. Therefore, within stable horse groups, aggressive behavior occurs only at low frequencies and in mild, ritualized forms^1,5,7,12,14,16,18,28–39^.

Horses’ social interactions are broadly divided into agonistic (socionegative), affiliative (sociopositive) and neutral behaviour^6^. Agonistic (aggressive and submissive) behaviours include biting, kicking, threatening, rump presentation, lungeing, avoidance or displacement^5,30^. Agonistic interactions result in immediate separation following a proximity event between two individuals to maintain or increase the interindividual distance (spontaneous displacement, walking away or fleeing)^5^. In contrast, during affiliative interactions, horses approach each other and typically remain within 1–2 body lengths of each other for extended periods^3,12,16,28,32,37,40–43^. Horses typically choose to associate with a small subset of the available group members, their preferred affiliative partners, with whom they spend time in spatial proximity when resting or feeding and participate in specific affiliative behaviours such as allogrooming^3,5,10,12,16,28,40^. Previous research has shown that affiliative interactions may have a calming and stress-reducing effect, as reflected by a lower heart rate and cortisol concentration after grooming, and contribute to social group stability, reproductive success, and equine welfare^12,28^. Given the significance of social interactions for horses, it is imperative to include social behaviour into welfare and quality of life assessment tools. To achieve this, it is necessary to establish objective, quantifiable, and evidence-based parameters that can accurately evaluate equine social interactions within the practical constraints of welfare assessments. Ideally, these parameters should also be applicable by horse owners, yard managers, and other stakeholders without extensive scientific training, facilitating widespread adoption and integration into real-world welfare assessments.

Proximity associations, encompassing both the closeness and duration of interactions, are considered good indicators for equine affiliative relationships, as only close companions are tolerated within a horse’s personal space and social bonds are characterized mostly by proximity^3,28,40,41,44^. In contrast, as agonistic interactions result in increasing distance between opponents, they can be identified by immediate separation following proximity. Thus, spatial relationships and dynamics as indicators of social behaviour and welfare^45^ can help optimize group composition and quality of life in horses by revealing affiliative social partners that should remain together, agonistic dyads that should be separated, and indicating withdrawn animals that may require additional attention, veterinary care and management optimization. Traditionally, such studies have been purely observational based on direct observation or analysis of video recordings, which is prone to observer bias, often limited to daylight hours and too time and resource-intensive to be feasible for welfare assessment^41,46^. Global positioning systems (GPS) are also of limited use for analyzing social behaviour due to their lacking functionality indoors and positional errors of 5–20 m^47^. New technological developments allow researchers to automatically collect absolute or relative spatial position data using ultra-wideband proximity sensors or tracking systems, continuously recording the precise location of all animals in the herd with a high temporal and spatial resolution^48–52^. This methodology is already well established in precision livestock farming for cows^47^ but has not yet been applied to the study of equine social proximity patterns. Therefore, this study aims to establish and validate the use of wearable ultra-wideband proximity sensors to quantify the spatial relationships and dynamics of social behaviour in horses.

## Material and methods

### Horses and horse management conditions

**Figure 1:**
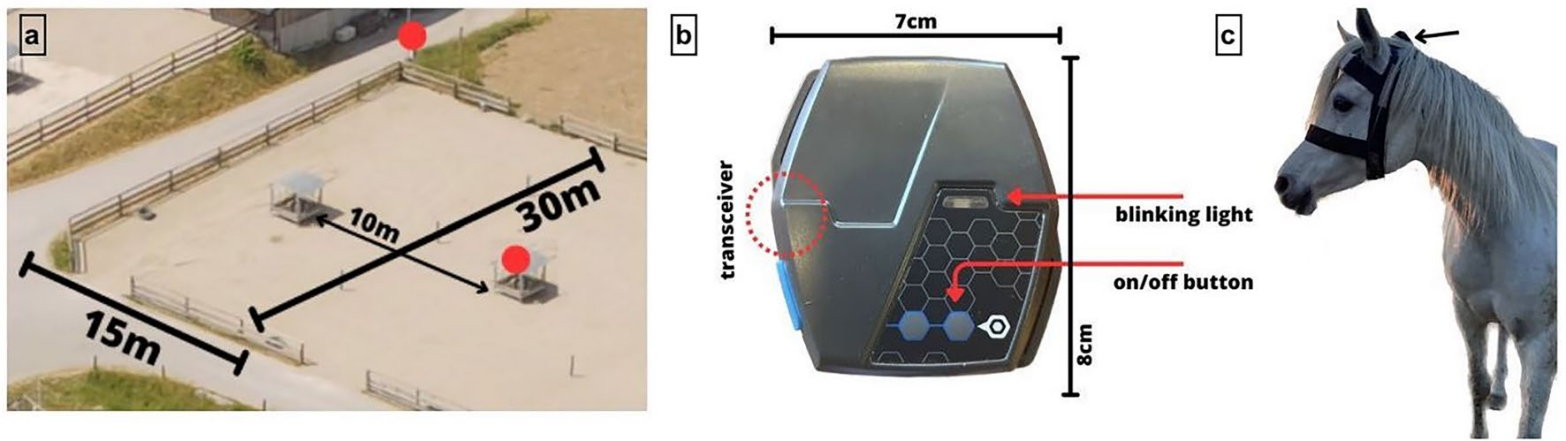
Experimental setup. (**a**) Image of the paddock (aerial photograph provided by Gut Aiderbichl), where the horses were turned out during the study. The dimensions of the paddock are marked in the image along the fence line, and the red dots represent the location of the video cameras, which were situated outside the paddock. (**b**) Image of the UWB proximity tag. The dimensions and the location of the transceiver, light and button are indicated in the image. (**c**) Photograph of a horse equipped with the UWB tag. The tag was attached to the horse's halter between the ears (indicated by the black arrow).

Eight mixed-breed horses, four geldings and four mares, aged 12–31 years (mean age 25 years ± 5.9 years s.d.), with an average body length of 153 cm (± 10 cm s.d.) were included in this study. The horses were located at an equine sanctuary and housed in individual box stalls, bedded with shavings, with daily paddock (450m2, 30 m x 15 m, Fig. 1a) turn-out for 4–6 h (appr. 07:30 am to 12:45 pm). The group composition was stable for at least two months; five horses were grouped together for seven years, one horse had joined the group one year ago and the last two horses three and two months ago, respectively. Horses had ad libitum access to water and were fed a hay‐based diet. In addition to the ad libitum access to hay provided in one hay feeder with eight feeding places on the paddock, all horses received two additional servings in the stable in the afternoon and at night, ensuring near continuous access to food throughout the day.

### Materials: wearable ultra-wideband proximity transceiver

An ultra-wideband (UWB) wireless real-time location system (RTLOC®) with a resolution of 1 measurement per second and a measurement range of a minimum of 5 cm and a maximum of 100 m was used for proximity tracking in this study. The UWB radiofrequency technology requires no fixed infrastructure. UWB devices join an ad-hoc network and start ranging. By measuring the time-of-flight (TOF) of UWB signals, the distance between two transceivers is obtained using the two-way-ranging (TWR) method, which calculates the distance between the nodes by multiplying the TOF by the speed of light. In this study, wearable UWB tags (8 cm × 7 cm × 2 cm, 95 g, Fig. 1b) that could easily be affixed to a horse’s halter and a gateway device to connect to a computer were used to continuously calculate and record the relative location of each tag.

### UWB proximity measurement accuracy under lab conditions

To test the accuracy of UWB distance measurements, two tags were placed at set reference distances (first 100 cm, then 200 cm, 300 cm, 500 cm, and 1000 cm) apart, and the inter-device distance measurements were recorded for 15 min for each distance (75 min in total, × 5 set distances, 15 min/distance). The deviation of the measurement distance of the two devices to the true distance (reference measure) was calculated using the following formula: *Deviation* =*|Measured Distance−True Distance|*, where: “*Deviation*” represents the difference between the measured distance and the true distance. “*Measured Distance*” refers to the distance obtained from the UWB measurements. “*True Distance*” indicates the actual reference distance between the two tags (measured using a ruler).

In addition, the temporal measurement stability and the influence of the spatial arrangement and transceiver orientation on distance measurements were evaluated by deploying seven tags at a 1 m distance around an eighth device, measuring the distance to the central eighth tag for 15 min, and calculating the difference between the actual and measured distance. The effect of transceiver orientation on distance measurements was further assessed by placing the seven tags next to each other at 1 m distance to the 8th device and measuring the distance for 60 min while changing the orientation of the 8th transceiver relative to the other seven tags every 15 min by 90°.

### UWB proximity measurements

The UWB tags were attached to the halter of each horse, between the ears (Fig. 1c), to measure interindividual distances. An additional tag was placed in the centre of the shared hay feeder to determine the horses’ proximity and thus access to the hay resource. The distances between individuals and the distance to the hay feeder were continuously measured for ten days (between November and December 2022) during paddock turn-out (appr. 5 h/day, total: 52 h 30 min). To exclude any erroneous measurements caused by a transient poor connection quality, interference by another horse moving between two transceivers or similar obstructions, all measurements greater than 5000 cm (not possible based on the paddock dimensions) and between 0 and 25 cm (impossible due to the sensor location behind and between the ears) were removed.

### Validation of the proximity data using video surveillance and video-based dyadic distance measurements under field conditions

Two cameras (GoPro HERO4, 1280 × 960p 60 fps) were installed to continuously record the interactions of the horses during paddock turn-out for sensor validation purposes, ensuring complete coverage of the paddock (Fig. 1). One was affixed to a pole at a height of 2 m, at an angle of 90° and a distance of 1.40 m to the hay feeder. The second camera was affixed on top of the hay feeder in the adjacent paddock at a height of 2.30 m, at an angle of 180° and a distance of 1 m to the focal hay feeder.

To determine the UWB measurement accuracy and convergent validity^53^ under dynamic conditions, timestamped UWB proximity data were collected simultaneously with timestamped video recordings and compared to video-based distance measurements. For the visual measurement, the single proximity tags were localized in the calibrated images^54^, where the intrinsic camera parameters are estimated for both cameras separately. The thus computed internal camera matrix allows us to remove the radial and tangential distortions. In this way, the required geometric properties required for a standard projective model (in particular in the context of radial distortions) are fulfilled. The real-world distances were estimated in a two-stage approach. In the first stage, the distance in pixels between these tags (assuming the central position) was estimated. In the second step, the pixel distances were mapped onto world distances. To this end, assuming that the tags were moving in a plane 1.8 m above the ground, the real-world distances were computed using a homography-based approach^55^. Indeed, from as few as four (more is better) known real-world coordinates within the assumed plane, a homography can be estimated, allowing us to map the real-world 3D distances to real-world 2D distances within the pane. Since this step is computed for each single view, the actual camera position (mounting height and angle) is not of relevance.

For verification, six sequences where the horses and the halter were visible and could be localized and identified reliably were selected and annotated. To demonstrate the accuracy, independently from the image localization, in this test set different scenarios were considered: horses moving just close to the camera, horses moving in at the maximum possible distance to the camera, horses moving in both scales (up to down, down to up, etc.). Taking a measurement every 10th frame, in total, 221 frames were annotated and used for validation.

### Social proximity measurements between horses

**Figure 2:**
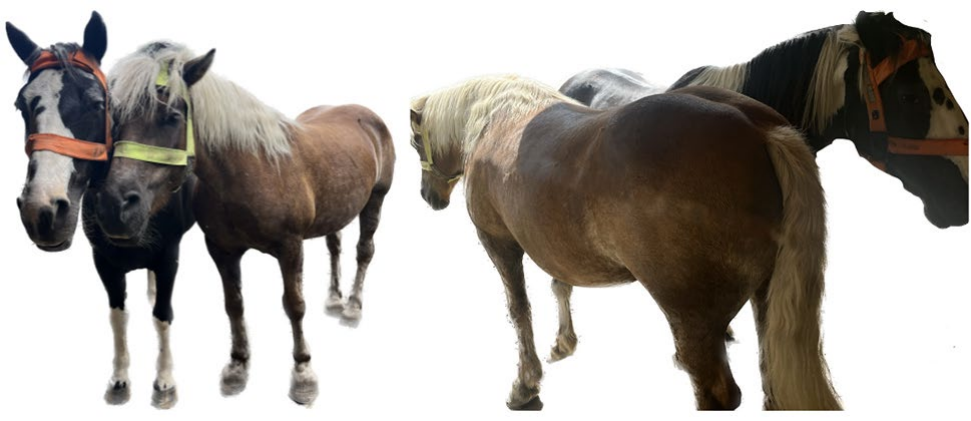
Photographs of an affiliative dyad standing in close proximity in two different configurations, illustrating the range of distance measurements that may indicate affiliative interactions. Specifically, in this example, the minimum distance between the two tags of the dyad configuration on the left is 25.5 cm (= (head-width horse 1 (25 cm) + head-width horse 2 (26 cm))/2) while the minimum distance of the dyad configuration on the right is 216.5 cm (= hypotenuse of a triangle, where side 1 = 210 cm (= average of the head–tail length of horse 1 (210 cm) and horse 2 (210 cm)), side 2 = 53 cm (= ½ average chest width of the two horses (= (40 cm + 50 cm)/4 = 22.5 cm) plus ½ the average pelvic width of the two horses (= (60 cm + 62 cm)/4 = 30.5 cm)).

The interindividual distance between all horse dyads while they were turned out together in a paddock was calculated for the 10-day observation period. In addition, the percentage of time spent at ≤ 1 m, 1–2 m, 2–3 m, 3–5 m, 5–10 m and further than 10 m apart was calculated by counting the measurements for each of these distance ranges and dividing this count by the total number of distance measurements. Based on the literature^3,40–43^ and to account for the different configurations of affiliative social interactions (Fig. 2), a cut-off of ≤ 3 m distance was used to define social proximity. Preferred partners were identified based on the percentage of time spent within an interindividual distance ≤ 3 m. Additionally, to establish a reference point for social proximity during affiliative grooming, the UWB distance measurements corresponding to 10 allogrooming episodes identified through video surveillance that lasted a minimum of 15 secs were analysed.

The speed of approach/retreat was calculated using the formula: speed = (distance[n]−distance[n−1])/(time[n]−time[n−1]), where distance[n] represents the distance measured at time point n (time[n]) and distance[n−1] represents the distance measured at the previous time point n−1 (time[n−1]). Speeds greater than 8 m/s were discarded as measurement errors based on the average velocity of horses at an extended canter of 6.4 m/s (+/− 0.26 s.d^56^) with a safety margin of 20% to avoid erroneous exclusion of data points. To calculate the speed of approach, only negative distance changes (= decreasing distance), and to calculate the speed of retreat, only positive distance changes were included. Ten video sequences of affiliative approaches (definition: Table 1^6,57–59^), eight (no further agonistic approaches were observed) video sequences of agonistic approaches and three canter episodes without interaction partner (no further canter episodes without interaction partner occurred) were identified and the corresponding UWB data analysed to determine the speed characteristics for these three movement patterns.

**Table 1:** Definitions of the social behaviours and social interaction characteristics included in this study.

Behaviour	Definition
Affiliative approach	Approach resulting in an interindividual distance ≤ 2 body lengths (3 m) for ≥ 10 s without resulting in agonistic interactions (modified from ^6,41,59^)
Agonistic approach	Approach resulting in retreat to maintain or increase the interindividual distance (modified from ^6,18,30,31,57^)
Retreat	Movement that maintains or increases a horse’s distance to an approaching conspecific (modified from ^6,30,60^)
Preferred partner	Conspecific with whom a horse spent most time in proximity ≤ 2 body lengths (3 m) (modified from ^28,61–63^)

### Proximity measurements between horses and the hay resource

To determine the distance of the horse-mounted tags to the device placed in the centre of the square hay feeder (dimensions: 2 m × 2 m) under the hay from which the horses were eating, 20 images extracted from video sequences were analysed, and the distance measurements of horses observed eating and of horses standing next to the hay feeder (not eating) were comparatively analysed. The proximity of the horses to the hay feeder and the time spent at specific distance ranges from the hay feeder was calculated analogously to the dyadic distance parameters.

### Statistical analysis

Statistical analysis was carried out with Graphpad Prism (version 9.5.1). As the data distribution of the distance measurements was determined to be non-Gaussian distribution by the D-Agostino & Pearson test, nonparametric analysis methods were applied, using an alpha of 0.05 for statistical significance. The correlation between all measurements of each dyadic tag pair (= technical replicates of the interdyadic distance measurements, e.g. distance measured between horse 1 and horse 2 by sensor 1 and sensor 2) and the correlation of UWB-proximity measurements with video analysis was calculated using the Spearman correlation coefficient test. Measurements of each dyadic tag pair (= technical replicates) were averaged for downstream analysis. The differences in distance measurements and speeds were analyzed using the Wilcoxon signed-rank test for paired data, such as the difference between actual and measured distances, and the Mann–Whitney U test or the Kruskal–Wallis test for unpaired data, such as the difference between horses eating vs standing next to the hayfeeder.

### Ethics approval

This study was non-invasive and entailed only monitoring the horses under their current conditions of life. No specific veterinary treatments or interventions were carried out for the purpose of this study. The study was reviewed by the Institutional Ethics Committee of the University of Veterinary Medicine Vienna (ETK-152/09/2019) in accordance with the “Good Scientific Practice. Ethics in Science and Research” guidelines implemented at the University of Veterinary Medicine Vienna and national legislation; ethical approval was waived.

## Results

### UWB proximity measurement accuracy under lab conditions

The median difference between the distance measured using the UWB tags and the actual distances (1 m, 2 m, 3 m, 5 m and 10 m) was 32 cm (95% confidence interval (CI): 31–32 cm, p < 0.0001, Table 2).

**Table 2:** Difference (median, 95% confidence interval (95% CI) and interquartile range (IQR)) between the actual distance and the distance measured using the UWB- sensors.

Actual distance (cm)	Measured distance (cm)		
	Median	95% CI	IQR
100	146	126–148	116.5–153
200	229	229–229	227–231
300	345	345–345	345.5–355
500	545	545–545	542.5–547
1000	1014	1014–1014	1011.5–1016.5

**Figure 3:**
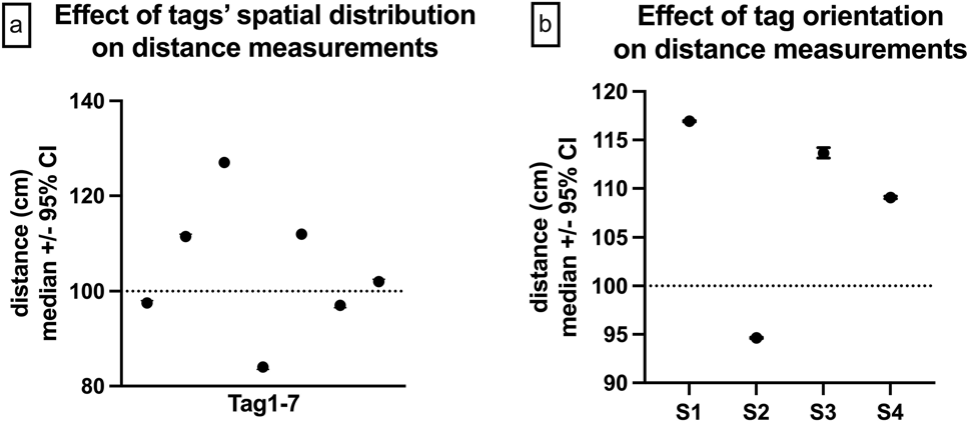
Effect of the spatial distribution of the UWB tags and the transceiver orientation on distance measurements. (**a**) Seven tags placed at a 1 m distance around an 8th device show significant measurement differences based on their spatial distribution (p < 0.0001). (**b**) The transceiver orientation (S1: pointing away from the other tags), toward the other devices (S2), and to the side (S3 and S4) of the other tags) significantly influenced distance measurements.

The spatial arrangement of the seven tags at a distance of 1 m around a central 8^th^ tag had a significant influence on distance measurements (p < 0.0001, Fig. 3). The measured median distance of the seven tags surrounding an 8th device at a distance of 100 cm was 102 cm (95% CI: 84-127 cm, interquartile range (IQR): 97–112 cm). The temporal stability of the measurements was good over the 15-min measurement period with a coefficient of variation < 3.3%.

Assessing the effect of transceiver orientation on distance measurements by turning the transceiver of one device every 15 min 90° relative to seven tags placed adjacent to each other at a distance of 1 m from the first revealed a significant effect of the orientation of the UWB transceiver (p < 0.0001, Fig. 3). The distance measurement was lowest with the transceiver pointing toward the other tags (median: 95 cm, 95% CI: 95–95 cm, IQR: 94–95 cm) and highest with the transceiver pointing away (median: 117 cm, 95% CI: 117–117 cm, IQR: 116–118 cm).

### UWB proximity measurements

All horses, accustomed to wearing halters, tolerated the wearable UWB tags well, and no attempts to remove the halter or the affixed tag or dermal irritations were observed. Data collection and transfer functioned well (no data was lost), and no technical problems were encountered during the 10-day observation period.

A total of 12,388,176 values, representing 1.049% of the entire dataset (continuous distance measurements for 10 days during turn-out at a resolution of 1 measurement/sec resulted in 1,180,951,000 data points), were excluded.

The high dyadic inter-device correlation (Spearman r = 0.98, 95% CI: 0.9773–0.9774, p < 0.0001, difference between medians: 3 cm) confirmed excellent technical reproducibility and accuracy and allowed using the average of the dyadic measurements (= technical replicates) for downstream analyses.

### Validation of the proximity data using video surveillance and video-based dyadic distance measurements under field conditions

Validation of UWB distance measurements using video analyses on a suitable representative subset yielded a correlation of 0.83, *p* < 0.0001, confirming the UWB’s convergent validity. In this case, “suitable representative” means that the sensors have been visible in the selected frames and the distances vary from close- to far-range, taking into account the complex geometry of the scene (i.e., a distance of one pixel correctly describes different distances in real-world).

### Social proximity measurements between horses

**Figure 4:**
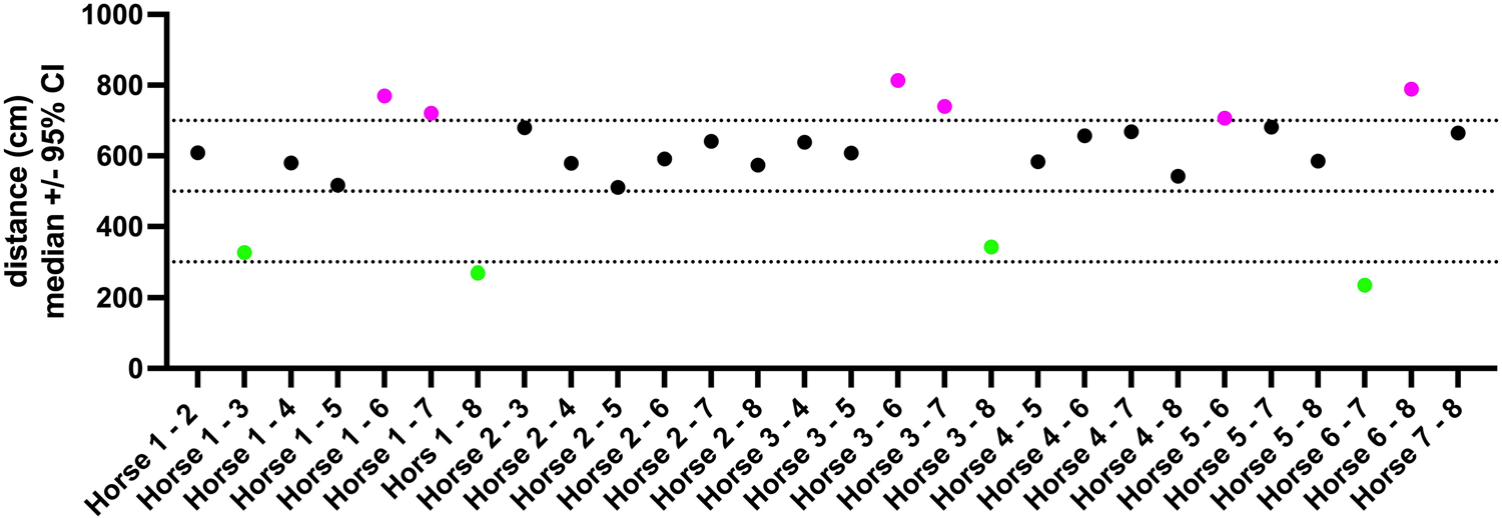
Horses’ interindividual distances, measured using UWB tags attached to the halter of each horse between the ears. Dotted lines at 3 m, 5 m and 7 m are provided as a reference frame. Horses which were close affiliates (the triad of horses 1, 3 and 8 and the dyad of horses 6 and 7) are shown in green; horses which remained a median of 7 m and further apart are indicated in pink.

Social proximity measurements using UWB tags affixed to the halter of each horse between the ears, revealed that the horses remained a median of 5.82 m (95% CI: 5.13–6.41 m) apart from each other (Table 3, Fig. 4) and spent only a median of 20% (95% CI: 14–26%, IQR: 14–27%) of their time in a distance ≤ 3 m (Table 4).

**Table 3:** Interindividual proximity (distance in cm, measured using the UWB tags affixed to the horses’ halters between the ears) with median, 25–75% Quartile (IQR) and 95% Confidence Interval of the median (95% CI).

		Horse 1	Horse 2	Horse 3	Horse 4	Horse 5	Horse 6	Horse 7	Horse 8
Horse 1	Median		609	327	580	517	770	721	270
	IQR		356–999	170–582	384–920	242–812	508–1275	426–1143	147–455
	95% CI		605–612	325–329	577–583	515–520	767–773	719–725	268–271
Horse 2	Median	609		681	579	511	592	641	574
	IQR	356–999		420–1120	345–883	254–760	275–1084	276–1021	269–1073
	95% CI	605–612		677–684	577–582	509–513	589–597	638–644	570–578
Horse 3	Median	327	681		639	608	813	740	343
	IQR	170–582	420–1120		436–944	381–926	540–1265	457–1104	223–558
	95% CI	325–329	677–684		636–641	606–611	809–817	737–743	341–344
Horse 4	Median	580	579	639		584	657	669	543
	IQR	384–920	345–883	436–944		352–908	434–1036	433–1015	332–924
	95% CI	577–583	577–582	636–641		581–587	655–659	666–672	540–545
Horse 5	Median	517	511	608	584		707	681	585
	IQR	242–812	254–760	381–926	352–908		443–1040	445–1038	352–871
	95% CI	515–520	509–513	606–611	581–587		705–710	678–683	583–588
Horse 6	Median	770	592	813	657	707		236	789
	IQR	508–1275	275–1084	540–1265	434–1036	443–1040		137–561	437–1303
	95% CI	767–773	589–597	809–817	655–659	705–710		235–238	786–793
Horse 7	Median	721	641	740	669	681	236		665
	IQR	426–1143	276–1021	457–1104	433–1015	445–1038	137–561		296–1127
	95% CI	719–725	638–644	737–743	666–672	678–683	235–238		662–668
Horse 8	Median	270	574	343	543	585	789	665	
	IQR	147–455	269–1073	223–558	332–924	352–871	437–1303	296–1127	
	95% CI	268–271	570–578	341–344	540–545	583–588	786–793	662–668

**Table 4:** Percent time (median, 95% confidence interval) the horses in this group spent in proximity to another horse.

Distance to other horses	Percent time spent at the distance	
	Median	95% CI
Less than 1	4.1	2.5–6.0
Between 1 and 2	7.9	5.9–9.7
Between 2 and 3	7.6	6.0–8.5
Between 3 and 5	19	17–21
Between 5 and 10	37	33–41
More than 10	25	20–28

**Figure 5:**
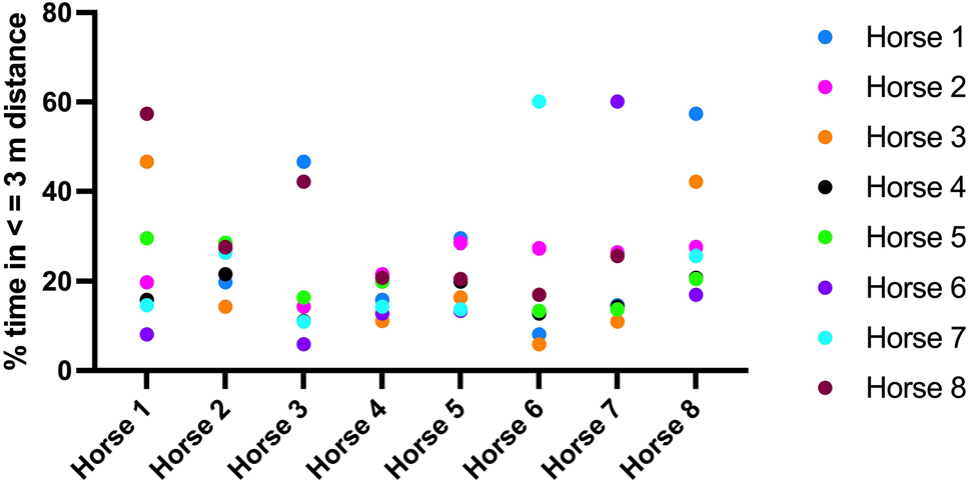
Time spent in proximity <  = 3 m. The affiliation of the triad of horses 1, 3 and 8 and the dyad of horses 6 and 7 is clearly evident. Horses 2, 4 and 5 (group leader) have no clear preferred associates.

For the individual horses, the preferred partner was at a distance ≤ 3 m for 21.59–60.11% of the time (Table 5, Fig. 5). The group of 8 horses was divided into one triad (horses 1, 3 and 8) and one dyad (horses 6 and 7) of close associates and three horses with no clear affiliative partner (horses 2, 4 and 5 (= group leader), max % time spent in ≤ 3 m to another horse: 29.46%). The preferred-partner-based grouping determined from the proximity data was in accordance with video surveillance and caretaker observations. During affiliative grooming, horses were a median of 74 cm apart (95% CI: 72–77, IQR: 58–90 cm).

**Table 5:** Percent time each horse spent in a distance ≤ 3 m to the other horses in the group.

	Horse 1	Horse 2	Horse 3	Horse 4	Horse 5	Horse 6	Horse 7	Horse 8
Horse 1		19.791	**46.647**	15.822	**29.48**	8.1294	14.622	**57.362**
Horse 2	19.791		14.294	**21.587**	28.51	27.289	26.354	27.56
Horse 3	46.647	14.294		11.095	16.399	5.883	10.956	42.14
Horse 4	15.822	21.587	11.095		19.881	12.798	14.300	20.79
Horse 5	29.48	**28.51**	16.399	19.881		13.36	13.695	20.491
Horse 6	8.129	27.289	5.883	12.7975	13.36		**60.111**	16.969
Horse 7	14.622	26.352	10.956	14.3002	13.695	**60.111**		25.657
Horse 8	**57.362**	27.56	42.138	20.7899	20.491	16.969	25.657	

For each horse, the time spent with its preferred partner is marked in bold. The affiliative triad of horses 1, 3, and 8 and the dyad of horses 6 and 7 spent most time in close proximity to their preferred partners. Horses 2, 4 and 5 spent < 30% of their time in close proximity to another specific horse.

Horses moved at a median speed of 38 cm/s (95% CI: 37.9–38.1 cm/s, IQR: 30.4–51.1 cm/s), equivalent to 1.37 km/h. The overall speed of approach (median 37 cm/s (= 1.33 km/h), 95% CI: 36.9–37.0 cm/s, IQR: 28.6–50.8 cm/s) was similar to the speed of separation (median 36.9 cm/s (= 1.33 km/h), 95% CI: 36.8–37.0 cm/s IQR: 28.6–50.5 cm/s).

**Figure 6:**
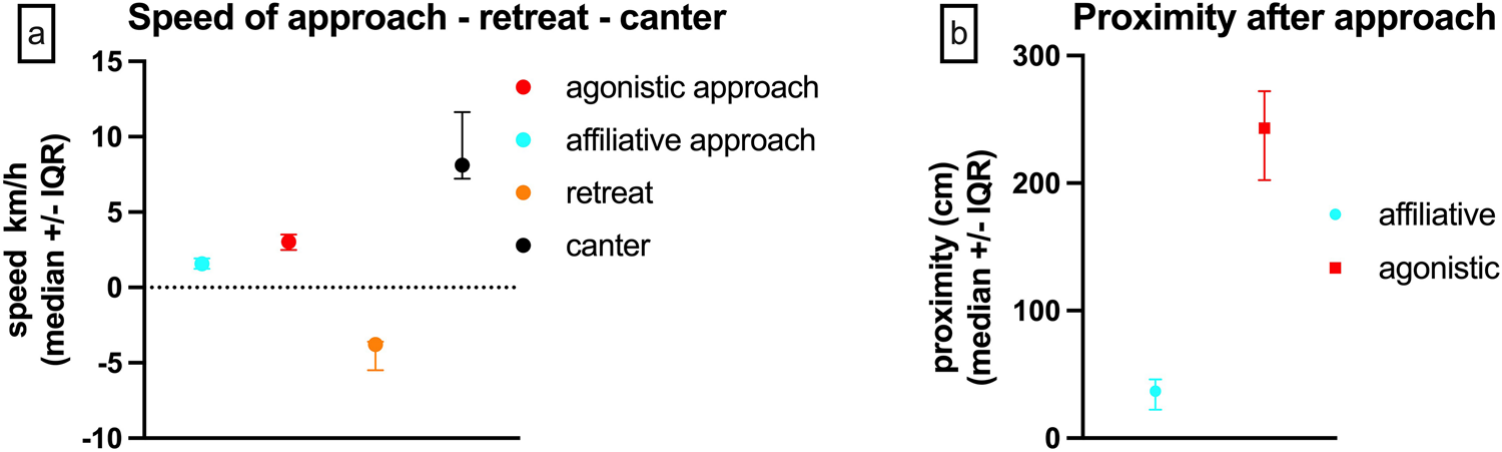
Differences between affiliative and agonistic approaches. (**a**) Speed of affiliative approach compared to agonistic approach, retreat after agonistic approach and canter without social interaction. (**b**) Greatest proximity following affiliative versus agonistic approach.

Affiliative approaches, characterized by a median speed of 1.57 km/h (95% CI: 1.26–1.92 km/h, IQR: 1.25–1.93 km/h) to a median proximity of 36.75 cm (95% CI: 19.5–62 cm, IQR: 20.6–50.4 cm) without retreat of the interaction partner (Fig. 6, suppl. video 1), were statistically significantly slower than agonistic approaches (p = 0.0394) and resulted in significantly greater proximity (p = 0.0003). Agonistic approaches occurred at a median speed of 3.04 km/h (95% CI: 2.16–3.74 km/h, IQR: 2.48–3.51 km/h) to a median proximity of 243 cm (95% CI: 130–319 cm, IQR: 198.1–272.4 cm) followed by an immediate retreat of the approached horse at a median speed of 3.77 km/h (95% CI: 3.52–5.85 km/h, IQR: 3.6–5.49 km/h, Fig. 6, suppl. video 2). Agonistic approaches were statistically significantly slower than retreat (p < 0.0001). In comparison, horses cantering without approaching a specific horse reached a median speed of 8.11 km/h (95% CI: 6.71–13.6 km/h, IQR: 7.21–11.6 km/h) and did not cause a retreat of the horses they passed, although they cantered past some horses at a distance ≤ 2 body lengths (suppl. video 3).

### Proximity measurements between horses and the hay resource

**Figure 7:**
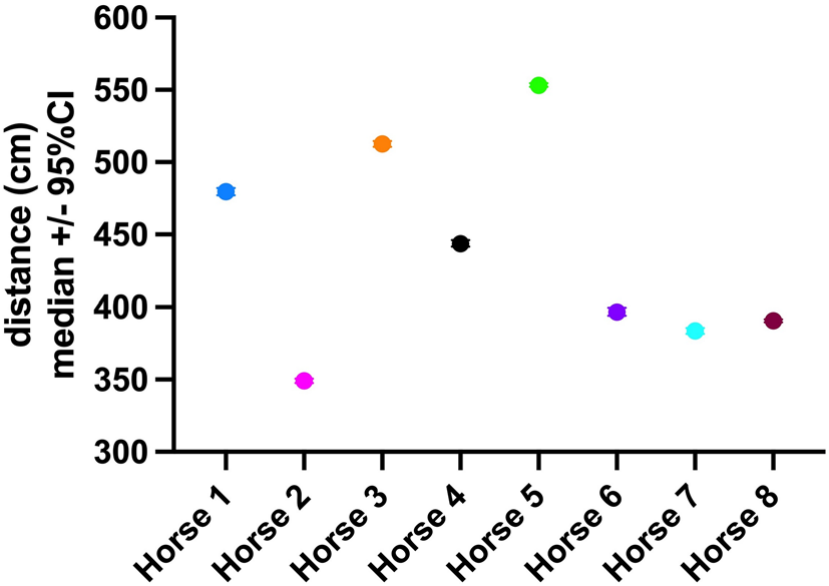
Distance of the individual horses from the hay feeder.

Horses remained at a median distance (measured between the tag placed in the center of the hay and the tags on the horses’ halters) of 4.46 m (95% CI: 4.45–4.47 m, IQR: 3.02–7.24 m) from the hay (Table 6, Fig. 7). The distance of horses to the hay sensor was significantly (p = 0.0182) lower while eating (median: 2.89 m, 95% Conf. Interval: 2.69–3.10 m, IQR: 2.49–3.16 m) than while standing next to the hay feeder without eating (median: 3.45 m, 95% CI: 2.68–4.15 m, IQR: 2.73–4.11 m). As the horses’ head could be a max. of 2.4 m from the tag placed in the center of the hay feeder while eating, dry tests were done, revealing the relatively large median distance measured between the horses’ tags and the hay tag to be caused by interference from the hay bale under which the tag was placed. Based on these data and video verification of eating at measured distances of 2.96 m, a cut-off of 3 m to the hay feeder was chosen to calculate the time the horses were eating. Horses in this group spent a median of 23% of their time (95% CI: 13–38%, IQR: 17–33%) in ≤ 3 m distance to the hay feeder. They approached the hay at a median speed of 42.5 cm/s (1.53 km/h, 95% CI: 42.5–43 cm/s, IQR: 29–67 cm/s) and moved away from the hay at a median speed of 42.5 cm/s (1.53 km/h, 95% CI: 42–42.5 cm/s, IQR: 28.5–66 cm/s).

**Table 6:** Distance (cm) of the individual horses from the hay resource.

Horse	Median	IQR	95% CI
Horse 1	480	334–758	477–482
Horse 2	349	260–696	348–351
Horse 3	513	367–823	511–515
Horse 4	444	301–624	442–446
Horse 5	553	341–715	552–555
Horse 6	397	270–850	394–400
Horse 7	384	283–695	382–386
Horse 8	391	309–791	390–392

## Discussion

This study demonstrates good technical reproducibility and accuracy of UWB distance measurements under both controlled laboratory and real-world field conditions based on the significant correlation of the UWB-proximity measurements with reference distances across varied spatial distributions and sensor orientations, the strong agreement between the interindividual distances recorded by the UWB sensors and those measured with video analysis and the high dyadic inter-sensor correlation. After testing the sensors’ spatiotemporal accuracy in a controlled static environment, field tests were conducted in a herd of eight horses during paddock turn-out to assess the validity, feasibility and utility of UWB proximity measurements under realistic conditions. In a herd environment in horses’ home enclosures, animals crossing between dyadic sensors and interference by other objects, such as hay feeders or stable walls can make visual tracking difficult. While UWB sensors, in contrast to visual methods, do not require line-of-sight, the radiofrequency signals they rely on can be significantly attenuated by water and hence bodies passing or standing between two tags can result in erroneously large distance readings^64–67^. Thus, validation of the UWB distance measurements for the particular complex conditions encountered in a horse herd is imperative.

The UWB data enabled identification of the preferred partners and calculation of the time spent in close proximity (≤ 3 m) to other horses as well as the speed of approach and retreat, confirming the utility of the UWB proximity measurements for the analysis of equine social behaviour. It's important to note that the halter-mounted UWB tags accurately measure distances between horses' heads, but may not reflect the closest proximity between individuals due to potential variations in body part positioning during social interactions. While proximity serves as a valuable metric, it is just one facet of the complex interactions that horses engage in and should be considered alongside other behavioral cues such as body language to gain a comprehensive understanding of equine social dynamics.

The horses large interindividual distances, the small percentage of time spent in close proximity (≤ 3 m) to a conspecific and the lack of an affiliative partner of three (out of eight) horses in this study suggest a suboptimal group composition, demonstrating the potential welfare applications of this technology.

Furthermore, the large interindividual distance and small percentage of time spent in close proximity to another horse measured in this study, raise the question of the distance thresholds for affiliative interactions versus departure from the group, especially considering the limited paddock dimensions. Traditionally one to two body lengths (1.5–3 m) are used to define horses’ personal space and distance range for association^3,4,9,41,42,68,69^, while a physical distance > 3 m from the closest group member is defined as departure from a group^70^. However, to date, research has relied on image- and video-based estimates of social proximity using horse length or GPS data with a measurement accuracy of ± 5 m. To our knowledge, horses' interindividual distances so far have never been measured with technology that allows measurement at cm level accuracy, such as UWB^41,71–74^. Therefore, to establish the association threshold measured by UWB technology and assess whether the measured distances reflect social interactions, this study determined the dyadic proximity measurements corresponding to allogrooming episodes identified on video surveillance. While, allogrooming was associated with close proximity (median distance of 74 cm) for a duration exceeding 15 s, these spatiotemporal parameters may also characterize other affiliative interactions^75,76^. Thus, further studies are needed to determine the distance and proximity duration thresholds for other social interactions to facilitate a more nuanced identification of different social behaviours by UWB technology.

Horses moved at a median speed of 38 cm/s (1.37 km/h), which, despite the older age of the horse population included in this study, compares well with previously published locomotion speeds during turnout of 0.7–1.16 km/h^77^. The speed of approach was similar to the speed of retreat, indicating that the social interactions of this group are not characterized by strong antagonistic events that would elicit rapid fleeing. The results demonstrate that the speed of approach can help distinguish between affiliative approaches and agonistic approaches. Affiliative approaches were slower (median 1.57 km/h) and resulted in greater median proximity (36.75 cm) than agonistic approaches (median 3.04 km/h, median proximity: 243 cm). As suggested in previous literature, agonistic approaches were followed by an immediate rapid retreat of the approached horse to increase or maintain the interindividual distance^59,61^, while horses remained in greater proximity after affiliative approaches. The retreat occurred faster (median 3.77 km/h) than the agonistic approach. To our knowledge, the speed of approach and retreat for affiliative and agonistic interactions has not been documented or reported in previous studies. These novel findings can aid in quantifying the occurrence of sociopositive and socionegative behaviours within horse groups, providing an additional tool for ethological research and contributing to the assessment and monitoring of the social aspect of equine welfare and quality of life.

The horses spent only 24.8% of their time within an eating distance (≤ 3 m) to the hay feeder, but their speed of withdrawal from the hay was similar to the speed of approach, suggesting that the hay resource might not be a source of conflict between the individuals of this group. It must be noted that the eating distance is based on the specific configuration of the hay feeder used in this study and would need to be adjusted for each feeding configuration. Similarly, the interindividual distance indicative of affiliative interactions may vary depending on the enclosure size and whether the horses are grazing or fed hay, requiring further studies to evaluate the influence of housing and management conditions on interindividual distances and proximity to resources.

In summary, the UWB technology yielded reliable and reproducible distance measurements under the real-world conditions of a group of horses that were turned out together. The utility of the proximity measurements for equine social behaviour research was demonstrated by the excellent accordance of affiliative partnerships (preferred partners) identified using UWB measurements with video surveillance observations. In addition, the proximity measurements and corresponding speed calculation allowed the identification of affiliative versus agonistic approaches based on differences in the speed of approach and the distance and duration of the resulting proximity, which may help identify suboptimal group composition and welfare problems based on the comparative prevalence of affiliative versus agonistic approaches. Furthermore, by placing a UWB sensor in the hay resource, this study could identify eating times based on proximity to the hay feeder and verify its correlation with feeding observed on video surveillance.

As affiliative interactions are characterized by close proximity of preferred associates, interindividual distance measurements can also serve as an objective and quantitative tool to monitor animal welfare and quality of life. For animal welfare applications, the suitability of the herd composition can be determined by analysing the amount of time spent within an affiliative distance range ≤ 3 m and the speed of approach and retreat. On an individual horse level, the availability of affiliative partners and access to resources can be evaluated. Since the types and frequencies of social interactions of domestic horses are influenced by the group composition, group stability, environmental and management conditions, including stocking density and access to resources, individual behaviour may vary over time and across situations and thus serve as a potential indicator of altered welfare and quality of life^12,28^. In addition, analogous to human patients^78^, sudden changes in social behaviour, increased aggressiveness, and withdrawal or isolation from environmental stimuli and the herd may indicate health problems or pain in horses^44^, inviting further research to study the effect of pain on equine social behaviour.

## Conclusion

Given the importance of sociality for horses, it is crucial to incorporate social behaviour into equine welfare and quality-of-life assessment tools. As affiliative interactions are mainly characterized by proximity, interindividual distance measurements can serve as an objective and quantitative indicator of equine social behaviour. The UWB technology validated in this study enabled continuous and accurate measurement of interindividual distances between all individuals in the group simultaneously, overcoming limitations of traditional observational methods. Evaluating affiliative distances and the speed of approach and retreat can determine individual affiliative partners and the suitability of herd composition. Since the social interactions of domestic horses are influenced by husbandry and management conditions, a change in social behaviour may indicate altered welfare and quality of life. Furthermore, withdrawal or isolation from the herd may indicate physical pain, warranting further research on the impact of health problems and environmental circumstances on equine social behaviour.

### Supplementary Information

Supplementary Video 1.

Supplementary Video 2.

Supplementary Video 3.

Supplementary Legends.

## Data Availability

The datasets generated and analysed during the current study are included in this published article (and its Supplementary Information files) or available from the corresponding author on reasonable request.
