# Supplementary material for: Proximity tracking using ultra-wideband technology for equine social behaviour research
Source: Sci Rep. 2024 Apr 30;14:9971. doi: 10.1038/s41598-024-60805-0 (PMC11063072; doi:10.1038/s41598-024-60805-0)
Supplement: Supplementary file 4 — Supplementary Legends. [file 41598_2024_60805_MOESM4_ESM.docx]

Supplementary Video 1: Affiliative approach

Affiliative approaches were characterized by a median speed of 1.57 km/h 95% CI: 1.26 – 1.92 km/h, IQR: 1.25-1.93 km/h) to a median proximity of 36.75cm (95% CI: 19.5-62cm, IQR: 20.6 – 50.4cm) without retreat of the interaction partner.

Supplementary Video 2: Agonistic approach

Agonistic approaches occurred at a median speed of 3.04 km/h (95% CI: 2.16 – 3.74 km/h, IQR: 2.48 – 3.51 km/h) to a median proximity of 243cm (95% CI: 130 – 319cm, IQR: 198.1 - 272.4 cm) followed by an immediate retreat of the approached horse at a median speed of 3.77 km/h (95% CI: 3.52 – 5.85 km/h, IQR: 3.6 – 5.49 km/h).

Supplementary Video 3: Horses cantering without approaching a specific horse

Horses cantering without approaching a specific horse reached a median speed of 8.11 km/h (95% CI: 6.71 – 13.6 km/h, IQR: 7.21 – 11.6 km/h) and did not cause a retreat of the horses they passed, although they cantered past some horses at a distance ≤ 2 body lengths.
